# The Impact of Comprehensive Genomic Profiling (CGP) on the Decision-Making Process in the Treatment of ALK-Rearranged Advanced Non-Small Cell Lung Cancer (aNSCLC) After Failure of 2^nd^/3^rd^-Generation ALK Tyrosine Kinase Inhibitors (TKIs)

**DOI:** 10.3389/fonc.2022.874712

**Published:** 2022-05-13

**Authors:** Ari Raphael, Amir Onn, Liran Holtzman, Julia Dudnik, Damien Urban, Waleed Kian, Aharon Y. Cohen, Mor Moskovitz, Alona Zer, Jair Bar, Natalie Maimon Rabinovich, Shirly Grynberg, Cecilie Oedegaard, Abed Agbarya, Nir Peled, Tzippy Shochat, Elizabeth Dudnik

**Affiliations:** ^1^ Department of Oncology, Tel-Aviv Sourasky Medical Center, Tel-Aviv, Israel; ^2^ Sackler Faculty of Medicine, Tel Aviv University, Tel Aviv, Israel; ^3^ Thoracic Oncology Service, Institute of Oncology, Sheba Medical Center, Ramat Gan, Israel; ^4^ Thoracic Oncology Service, Cancer Institute, Soroka University Medical Center, Beer-Sheva, Israel; ^5^ Department of Oncology, Shaare Zedek Medical Center, Jerusalem, Israel; ^6^ Thoracic Cancer Service, Rambam Health Care Campus, Haifa, Israel; ^7^ Thoracic Cancer Service, Meir Medical Center, Kfar Sava, Israel; ^8^ Department of Oncology, Bnai Zion Medical Center, Haifa, Israel; ^9^ Faculty of Health Sciences, Ben Gurion University of Negev, Beer-Sheva, Israel; ^10^ Statistical Consulting Unit, Rabin Medical Center, Petah Tikva, Israel; ^11^ Thoracic Oncology Service, Assuta Medical Centers, Tel-Aviv, Israel; ^12^ Thoracic Oncology Service, Rabin Medical Center, Petah Tikva, Israel

**Keywords:** comprehensive genomic profiling, next-generation sequencing, ALK, failure of ALK TKI, acquired resistance, decision impact

## Abstract

**Background:**

The use of CGP in guiding treatment decisions in aNSCLC with acquired resistance to ALK TKIs is questionable.

**Methods:**

We prospectively assessed the impact of CGP on the decision-making process in ALK-rearranged aNSCLC patients following progression on 2^nd^/3^rd^-generation ALK TKIs. Physician’s choice of the most recommended next-line systemic treatment (NLST) was captured before and after receival of CGP results; the percentage of cases in which the NLST recommendation has changed was assessed along with the CGP turnaround time (TAT). Patients were divided into groups: patients in whom the NLST was initiated after (group 1) and before (group 2) receival of the CGP results. Time-to-treatment discontinuation (TTD) and overall survival (OS) with NLST were compared between the groups.

**Results:**

In 20 eligible patients (median [m]age 63 years [range, 40-89], females 75%, adenocarcinoma 100%, failure of alectinib 90%, FoundationOne Liquid CDx 80%), CGP has altered NLST recommendation in 30% of cases. CGP findings were as follows: ALK mutations 30% (l1171X 10%, G1202R, L1196M, G1269A, G1202R+l1171N+E1210K 5% each), CDKN2A/B mutation/loss 10%, c-met amplification 5%. CGP mTAT was 2.9 weeks [IQR, 2.4-4.4]. mTTD was 11.3 months (95% CI, 2.1-not reached [NR]) and 5.4 months (95% CI, 2.0-NR) in groups 1 and 2, respectively (p-0.34). mOS was 13.2 months (95% CI, 2.9-NR) and 13.0 months (95% CI, 6.0-NR) in groups 1 and 2, respectively (p-0.86).

**Conclusion:**

CGP has a significant impact on the decision-making process in ALK-rearranged aNSCLC following progression on 2^nd^/3^rd^-generation ALK TKIs.

## Background

Approximately 3-5% of tumors in patients with advanced non-small cell lung cancer (aNSCLC) harbor rearrangements of the anaplastic kinase lymphoma gene (ALK) (1, 2). This is a unique aNSCLC subpopulation that mostly consists of young individuals, with no or limited history of smoking, and an adenocarcinoma histology. Although ALK fusion is a rare phenomenon, it shouldn’t be neglected considering the high prevalence of lung cancer overall, and the availability of several effective targeted treatment options (3–5).

The presence of ALK rearrangement results in tumor susceptibility to ALK tyrosine kinase inhibitors (ALK TKIs) (6). Crizotinib, a TKI of ALK, tyrosine-protein kinase Met (c-met), and ROS proto-oncogene 1 (ROS1) kinases (7), was the first ALK inhibitor to replace the standard chemotherapy in the 1^st^-line treatment of aNSCLC harboring an ALK fusion, providing the significant advantage of this therapy in terms of the progression-free survival - according to the results of the PROFILE 1014 trial (8). Since then, newer 2^nd^- and 3^rd^-generation ALK TKIs (e.g., alectinib, ceritinib, brigatinib, ensartinib and lorlatinib) were implemented into the management of ALK- rearranged aNSCLC - first in the post-progression setting (9–11), and later on - in the 1^st-^ line setting - that based on the results of the ALEX trial (12), the ALTA-1L trial (13), the ASCEND-4 trial (14), and the CROWN trial (15).

The questions of ALK TKIs sequencing and optimal treatment strategy following the disease progression on specific ALK TKIs remain open, since these have never been evaluated in a randomized controlled clinical trial (16). Treatment decisions, however, can be guided by the acquired resistance mechanisms responsible for the disease progression during systemic treatment. The mechanisms of acquired resistance to ALK TKIs primarily include development of secondary resistant mutations in the ALK kinase domain occurring in 25-66% of patients (17–21). Of those, G1202R/del mutations predominate (42-53% of cases), while other ALK mutation types responsible for the development of secondary resistance to ALK TKIs are: L1196M, F1174X, G1269A, L1196M, and I1171X (18, 21). Moreover, sequential treatment with increasingly potent ALK TKIs may promote acquisition of treatment-refractory compound ALK mutations (21, 22). Off-target mechanisms of resistance to ALK TKIs involve up-regulation of bypass signaling pathways, such as epidermal growth factor receptor (EGFR), c-met, KIT proto-oncogene (KIT), insulin-like growth factor 1 receptor (IGF-1R), proto-oncogene SRC (SRC), MEK/ERK and others (17, 18, 23, 24). SCLC transformation has been described as a resistance mechanism to ALK TKIs as well (25, 26).

Since ALK resistance mutations appear to be the predominant mechanism of resistance to ALK TKIs, there is a clear rationale for its targeting. Moreover, it has been demonstrated that the presence of an ALK resistant mutation following the progression on 1^st^- and 2^nd^-generation ALK TKIs in ALK-rearranged aNSCLC is associated with better lorlatinib efficacy (19). However, different 2^nd^- and 3^rd^-generation ALK TKIs appear to have different *in vitro* activity against specific ALK resistant mutations, which, therefore, represents the rationale for identifying the underlying ALK resistant mutation subtype before making the decision regarding the next line of systemic treatment.

In our study, we prospectively assessed the impact of comprehensive genomic profiling (CGP) on the decision-making process in patients with ALK-rearranged aNSCLC following progression on 2^nd^- and 3^rd^-generation ALK TKIs.

## Methods

### Patient Selection, Study Design and Assessments

**Figure 1:**
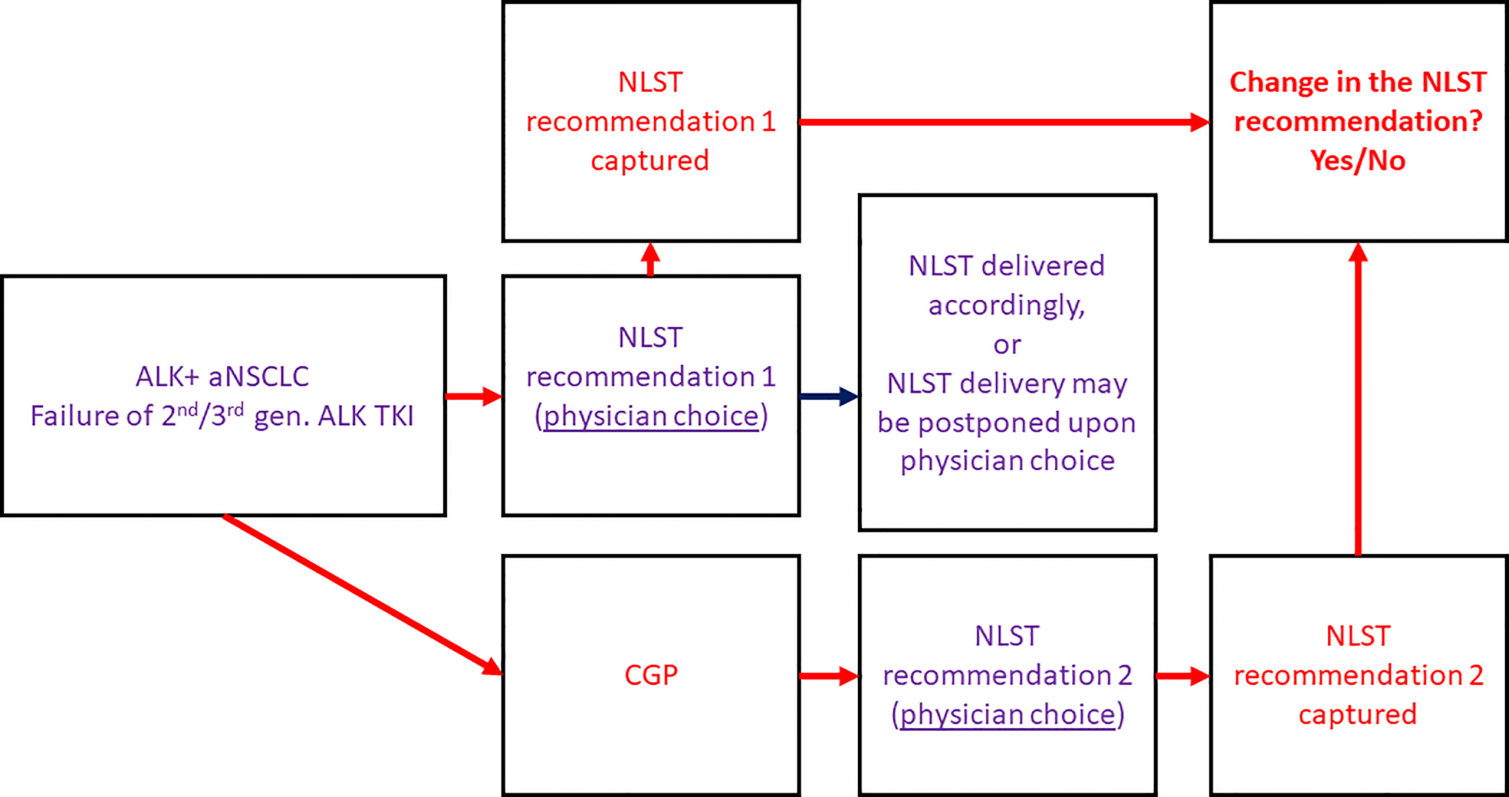
Study design. ALK, anaplastic lymphoma kinase; aNSCLC, advanced non-small cell lung cancer; CGP, comprehensive genomic profiling; gen., generation; NLST, next-line systemic treatment; TKI, tyrosine kinase inhibitor.

ALK-rearranged aNSCLC patients following failure of a 2^nd^/3^rd^-generation ALK TKI, regardless of prior crizotinib or platinum-based chemotherapy, treated in one of the participating Israeli oncological centres were selected for this prospective multicentre non-interventional clinical study. CGP [either in the form of FoundationOne CDx or FoundationOne Liquid CDx using algorithm as previously described in detail (27)] was performed, and the results were captured. A questionnaire was filled by the treating oncologist twice: before and after receival of the CGP results. The questionnaire (**Supplementary Document S1**) included the de-identified clinical patient data, the de-identified next-generation sequencing (NGS) results, and the physician’s choice of the most recommended next-line systemic treatment (NLST) captured before and after receival of CGP results. The percentage of cases in which the treatment recommendation has changed upon the receival of CGP results was assessed - reflecting the impact of the molecular testing on the decision-making process (**Figure 1**). We hypothesized that change in the treatment recommendation will occur in at least 30% of cases (the minimal clinically meaningful rate according to our perception, the cut-off was chosen arbitrarily).

We prospectively gathered an information regarding the CGP turnaround time (TAT). The number of patients with adverse outcomes while waiting for the NGS results was collected as well. Additional demographic and clinical patient data were retrospectively retrieved from the patient medical records at each of the participating Israeli oncological centers.

The decision regarding the NLST type and initiation was done by the treating oncologist and was not specified by the protocol (**Figure 1**). Therefore, there were patients in whom the NLST was initiated after receival of the CGP results and in accordance with the NGS findings (group 1), and patients in whom the treatment was initiated before the CGP results became available (group 2). Time-to-treatment discontinuation (TTD) and overall survival (OS) with the NLST were retrospectively assessed and compared between the groups.

Next, we selected ALK-rearranged aNSCLC patients following failure of alectinib or ceritinib (the most commonly used 1^st^ line ALK TKIs), regardless of prior platinum-based chemotherapy, and retrospectively assessed TTD with brigatinib and lorlatinib (the drugs typically used in this clinical scenario) in correlation with the NGS findings.

### Statistical Analysis

The sample size was determined by the available patients meeting the inclusion criteria and referred for CGP. The statistical analysis was generated using SAS Software, version 9.4 (28). Categorical variables were presented by numbers and percentiles, medians and ranges were reported for continuous variables. TTD and OS were assessed by the Kaplan-Meier method, with the log-rank test for the comparison. Two-sided p values less than 0.05 were considered statistically significant.

### Ethical Aspects

Institutional review board approval has been received before study initiation. No patient-identifying data was included in the central data collection.

## Results

### Patient Baseline and Treatment Characteristics

Twenty-two ALK-rearranged aNSCLC patients performed CGP within the study. One patient did not meet the eligibility criteria (failure of crizotinib and no administration of 2^nd^/3^rd^-generation ALK TKIs before enrolment), and the questionnaire was not filled by the treating oncologist in one additional patient – these two were excluded from the analysis. The baseline and treatment characteristics of the selected cohort (n=20) are presented in **Table 1**.

**Table 1:** Patient baseline and treatment characteristics in the whole study population and according to whether NLST was initiated before (group 1) or after (group 2) receival of CGP results.

	Patients according to group assignment (n = 16)*			All patients (n = 20)
	Group 1 (n = 8)	Group 2 (n = 8)	p value	
Age, years – median (range)	62 (40-68)	62 (50-84)	0.17	63 (40-89)
Sex, n (%)			1.00	
Female	5 (63)	6 (75)		15 (75)
Male	3 (37)	2 (25)		5 (25)
Smoking history, n (%)			1.00	
Current/past smoker	4 (50)	4 (50)		8 (40)
Never smoker	4 (50)	4 (50)		10 (50)
NA				2 (10)
Tumor histology, n (%)				
Adenoca	8 (100)	8 (100)	1.00	20 (100)
ECOG PS, n (%)			1.00	
0/1	5 (62.5)	5 (62.5)		11 (55)
2/3/4	2 (25)	2 (25)		4 (20)
NA	1 (12.5)	1 (12.5)		5 (25)
Brain metastases, n (%)	6 (75)	2 (25)	0.13	8 (40)
Previous ALK TKIs, n (%)			0.51	
Alectinib	6 (75)	8 (100)		18 (90)
Ceritinib	2 (25)	1 (12.5)		3 (15)
Brigatinib	3 (37)	1 (12.5)		4 (20)
Ensartinib	1 (12.5)	0 (0)		1 (5)
Lorlatinib	1 (12.5)	1 (12.5)		3 (15)
Crizotinib	3 (37)	4 (50)		7 (35)
Number of previous lines of ALK TKIs - median (range)	1 (1-4)	1 (1-4)	0.83	1 (1-4)
Previous platinum-based chemotherapy, n (%)	2 (25)	2 (25)	1.00	4 (20)
CGP type, n (%)			1.00	
FoundationOne Liquid CDx	7 (87.5)	7 (87.5)		16 (80)
FoundationOne CDx	1 (12.5)	1 (12.5)		4 (20)
ALK mutation, n (%)			0.43	6 (30)
G1202R	0 (0)	1 (12.5)		1 (5)
l1171X	0 (0)	1 (12.5)		2 (10)
L1196M	0 (0)	1 (12.5)		1 (5)
G1269A	0 (0)	1 (12.5)		1 (5)
Complex ALK mutation (G1202R, l1171N, E1210K)	1 (12.5)	0 (0)		1 (5)
Other potentially targetable aberrations	2 (25)	0 (0)		3 (15)
Presence of original ALK fusion, n (%)	5 (63)	4 (50)	1.00	11 (55)
NLST, n (%)			0.19	
Brigatinib	2 (25)	4 (50)		6 (30)
Lorlatinib	4 (50)	1 (12.5)		5 (25)
Platinum-based chemotherapy	1 (12.5)	2 (25)		3 (15)
Other	1 (12.5)	1 (12.5)		2 (10)
NA				4 (20)*
Reason for stopping NLST, n (%)			0.44	
Disease progression	1 (12.5)	2 (25)		3 (15)
Death	4 (50)	3 (37.5)		7 (35)
NA				4 (20)*
NLST ongoing, n (%)	3 (37.5)	3 (37.5)		6 (30)

*One patient did not initiate NLST at the time of this report, one patient died before getting any further systemic treatment, and the information regarding NLST is missing for two additional patients.

Adenoca, adenocarcinoma; ALK, anaplastic kinase lymphoma; CGP, comprehensive genomic profiling; ECOG PS, Eastern Cooperative Oncology Group performance status; NA, not available/not applicable; NLST, next-line systemic treatment; TKIs, tyrosine kinase inhibitor(s).

The median age of the patients in the cohort was 63 (range, 40-89); females and never smoking patients with adenocarcinoma histology predominated - as expected for the enrolled population. The majority of patients received alectinib (with or without crizotinib) before enrolment; four, three, three, and one patient, respectively, were treated by brigatinib, ceritinib, lorlatinib and ensartinib; four patients received platinum-based chemotherapy before enrolment.

### CGP Results and Change in Treatment Recommendation Upon Their Receival

FoundationOne Liquid CDx was the predominant CGP type performed. The molecular alterations diagnosed by NGS are presented in **Table 1**. ALK resistant mutations were present in 6 (30%) of cases (of those, G1202R in 1 case, l1171X in 2 cases, L1196M in 1 case, G1269A in 1 case, and a complex mutation in ALK gene combining G1202R, l1171N, and E1210K mutations in 1 case). With regards to another potentially targetable genomic aberrations, high level of c-met amplification was present in 1 case, and a cyclin dependent kinase inhibitor 2A/B (CDKN2A/B) mutation or loss was present in 2 additional cases. The original ALK fusion was presented in 11 (55%) of cases.

**Figure 2:**
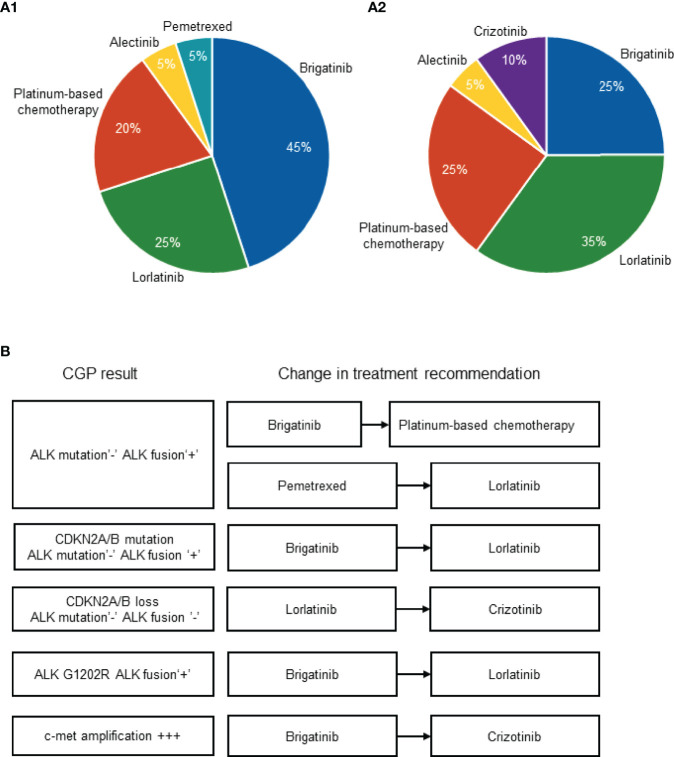
Physician’s choice of the most recommended NLST captured before **(A1)** and after **(A2)** the receival of CGP results. Change in treatment recommendation upon the receival of CGP results **(B)**. *ALK,* anaplastic lymphoma *kinase;* CDKN2A/B, cyclin dependent kinase inhibitor 2A/B; CGP, comprehensive genomic profiling; c-met, tyrosine-protein kinase Met; NLST, next-line systemic treatment.

Overall, the change in NLST recommendation upon receival of the CGP results was registered in 6 patients (30% of the patients in the cohort). The initial physician’s choice of the most recommended NLST captured before receival of NGS results was as follows: brigatinib, n=9 (45%); lorlatinib, n=5 (25%); platinum-based chemotherapy, n=4 (20%), alectinib, n=1 (5%); pemetrexed, n=1 (5%) (**Figure 2A**). The physician’s choice of the most recommended NLST captured after receival of NGS results was as follows: brigatinib, n=5 (25%); lorlatinib, n=7 (35%); platinum-based chemotherapy, n=5 (25%), alectinib, n=1 (5%); crizotinib, n=2 (10%) (**Figure 2A**).

The change in the physician’s recommendation occurred upon the diagnosis of the following molecular alterations: absence of ALK resistant mutation and presence of original ALK fusion, n=2 (which drove the switch from brigatinib to platinum-based chemotherapy in once case and the switch from pemetrexed to lorlatinib in another case); CDKN2A/B mutation, absence of ALK resistant mutation and presence of original ALK fusion, n=1 (which drove the switch from brigatinib to lorlatinib); CDKN2A/B loss, absence of ALK resistant mutation or original ALK fusion, n=1 (which drove the switch from lorlatinib to crizotinib); presence of ALK G1202R and presence of original ALK fusion, n=1 (which drove the switch from brigatinib to lorlatinib); high level of c-met amplification, n=1 (which drove the switch from brigatinib to crizotinib) (**Figure 2B**).

### CGP TAT

The median NGS testing TAT was 2.9 weeks [Interquartile range (IQR), 2.4-4.4]. One patient has died while waiting for the NGS results.

### Time-To-Treatment Discontinuation and Overall Survival With the NLST

The NLST was initiated after receival of the CGP results and in accordance with the NGS findings in 8 patients (group 1), and included: brigatinib, n=2; lorlatinib, n=4; crizotinib, n=1; and platinum-based chemotherapy, n=1. The NLST was initiated before the NGS results became available in 8 patients (group 2), and included: brigatinib, n=4; lorlatinib, n=1; alectinib, n=1; and platinum-based chemotherapy, n=2 (**Table 1**). In addition, one patient did not initiate NLST at the time of this report, one patient died before getting any further systemic treatment (the same patient described in the previous section), and the information regarding the NLST is missing for two additional patients.

The baseline and treatment characteristics according to group assignment are presented in **Table 1**. Higher proportion of patients in group 1 had brain metastases and had previous exposure to novel 2^nd^-generation ALK TKIs (e.g., brigatinib and ensartinib). Patients in group 2 were less frequently approached with lorlatinib; higher proportion of patients in group 2 appeared to harbor an ALK resistant mutation. Those differences were not statistically significant.

Importantly, the change in NLST recommendation upon receival of NGS results was registered in 4 out of 8 patients included in group 1 (50%); these included cases #1, #2, #3, and #4 (**Figure 2B**). Case #5 was included in group 2, and the information regarding the NLST is missing in case #6 (**Figure 2B**).

**Figure 3:**
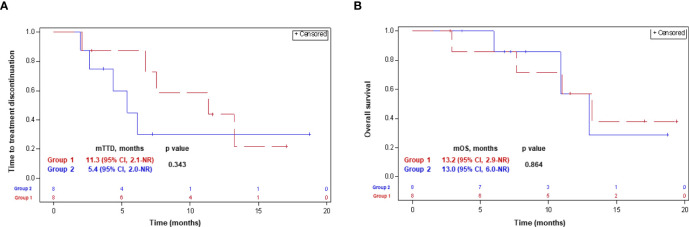
Time-to-treatment discontinuation **(A)** and overall survival **(B)** with the NLST in patients in whom the treatment decision was made before (group 2) and after (group 1) getting the CGP results. CGP, comprehensive genomic profiling; CI, confidence interval; NLST, next-line systemic treatment; NR, not reached; (m)OS, (median) overall survival; (m)TTD, (median) time-to-treatment discontinuation.

The median follow-up was 11.3 mounts [IQR, 5.3-15.1] and 7.8 months [IQR, 6.4-11.9] in groups 1 and 2, respectively. Five (62.5%) patients in each group discontinued the NLST at the time of the last follow-up. Four (50%) patients in group 1, and 3 (37.5%) patients in group 2, respectively, have died (**Table 1**). Median TTD was 11.3 months (95% CI, 2.1-not reached [NR]) and 5.4 months (95% CI, 2.0-NR) in groups 1 and 2, respectively (p-0.34). Median OS was similar in both groups, and comprised 13.2 months (95% CI, 2.9-NR) and 13.0 months (95% CI, 6.0-NR) in groups 1 and 2, respectively (p-0.86). The Kaplan-Meyer curves for the TTD and OS with the next-line systemic treatment according to group assignment are presented in **Figure 3**.

### Time-To-Treatment Discontinuation and Overall Survival With Brigatinib and Lorlatinib in Correlation With the NGS Findings

In one patient diagnosed with a complex G1202R, l1171N, and E1210K ALK mutation, TTD and OS with lorlatinib were 13.0 months and 13.0 months, respectively. In one patient diagnosed with a G1202R ALK mutation, TTD and OS with brigatinib were 2.0 months and 6.0 months, respectively. In one patient diagnosed with an l1171T ALK mutation, TTD and OS with brigatinib were 4.5 months and 8.0 months, respectively. One patient diagnosed with an L1196M ALK mutation, continues lorlatinib at the time of the report for 8.0 month since treatment initiation.

## Discussion

This is the first prospective study illustrating the value of CGP and molecular assessment of acquired resistance mechanisms in treatment decision-making process in ALK-rearranged aNSCLC patients.

According to our observation, CGP performed at the time of progression on 2^nd^- and 3^rd^-generation ALK TKIs, has altered treatment recommendation in one third of cases - which confirmed the initial hypothesis and, overall, appeared to be a clinically meaningful result. In cases the initiation of the NLST was postponed until getting NGS results, the proportion of patients in whom the treatment recommendation has changed was even higher (50%) - pointing to potentially larger effect of genomic assessment on the decision-making process.

Importantly, the median CGP TAT was only 2.9 weeks [IQR, 2.4-4.4] which seems acceptable considering the CGP impact on treatment decision. In our cohort, only one patient has died while waiting for the CGP results. This fact emphasizes the need to assess further the phenomenon of clinical deterioration attributable to rapid disease progression, and the potential adverse effect of the CGP in this association.

Looking into the specific treatment recommendation changes following the receival of the CGP results, we observed increase in the proportion of recommendations on lorlatinib, platinum-based chemotherapy, and crizotinib. There were three clinical scenarios which seemed to have a biologic rationale behind the treatment decision alteration. The 1^st^ clinical scenario was switching to a different targeted treatment following the diagnosis of another potentially targetable molecular aberration, or a bypass pathway activation, such as switching from ALK TKI to c-met TKI following the diagnosis of c-met amplification. Indeed, c-met alterations represent one of the most common mechanisms responsible for acquired resistance to osimertinib in EGFR mutant aNSCLC (10-25%) (29), and to ALK TKIs in ALK-rearranged aNSCLC (15%) (24). Moreover, c-met inhibition has been associated with objective response rate of 30% and median duration of response of 7.9 months in c-met-amplified aNSCLC patients following progression on 3^rd^-generation EGFR TKIs (30). Additionally, there are several case reports suggesting that met-inhibition may overcome c-met-driven resistance in ALK-positive aNSCLC (24, 31, 32). The 2^nd^ clinical scenario of treatment decision alteration in our cohort was switching from brigatinib to lorlatinib following the diagnosis of ALK G1202R mutation – which is justified by the high activity of lorlatinib in tumors harboring this ALK resistant mutation (19). The 3^rd^ clinical scenario in our study included switching from ALK TKIs to platinum-based chemotherapy in the absence of ALK resistant mutation - which, again, seems reasonable considering modest next-generation ALK TKI activity in patients without ALK resistant mutations following progression on prior ALK TKIs (19). Specifically, a positive correlation between presence of ALK resistant mutations, their type, and outcomes with lorlatinib have been reported in ALK-rearranged aNSCLC patients following failure of a 2^nd^-generation ALK TKI. It remains unknown, however, whether similar correlation is true for brigatinib. Moreover, no comparative clinical studies have been done or planned to be done in order to explore the comparative efficacy of the two agents in correlation with the molecular biomarkers in this clinical setting.

In three additional clinical scenarios which prompted treatment decision changes in our study, it was hard to explain the physician’s decision from the biologic perspective. For instance, presence of CDKN2A/B loss or mutation was anticipated to alter the decision towards cyclin-dependent kinase 4/6 (CDK4/6) inhibitors, however, it was not the case. Having said that, it should be emphasized that CDKN2A/B alterations are only rarely seen following progression on 2^nd^/3^rd^-generation ALK TKIs (17, 23, 33), and the majority of CDK4/6 inhibitors did not demonstrate a significant antitumor activity in aNSCLC (34–39). Although CDK4/6 inhibitors have demonstrated a myelo-preserving effect in conjunction with chemotherapy in advanced small-cell lung cancer, it did not appear to improve tumor control (40).

The prevalence of ALK resistant mutations (30%) and their distribution in our study were in line with the previously reported data in ALK-positive aNSCLC following treatment with 2^nd^/3^rd^-generation ALK TKIs (19, 23). Only one case of high-level c-met amplification was present in our cohort, while higher prevalence of c-met alterations (12-22%) has been reported in the literature (24). This discrepancy might be attributable to the lower proportion of patients progressing on lorlatinib in our cohort, and less so - to the technical limitations of the liquid biopsy assay. For instance, Dagogo-Jack et al. reported on higher prevalence of c-met amplification following treatment with 3^rd^-generation ALK TKI, on one hand, and on the other hand - on high overall accuracy of liquid NGS as compared to tissue genotyping (24). The detection of CDKN2A/B alterations was not unique to our cohort either (33). Another interesting observation in our study was tissue versus liquid biopsy referral patterns. For instance, liquid biopsy was the preferred method of assessment - probably due to its simplicity and high patient advocacy, which reflected real-world physician and patient preferences. This pattern was also in line with the recently updated International Association for the Study of Lung Cancer (IASLC) guideline on liquid biopsy to discover molecular resistance mechanisms (41).

CGP performed at the time of progression on 2^nd^/3^rd^-generation ALK TKIs demonstrated a positive impact on NLST duration but did not affect the OS. Several factors might have attributed to that. First, some of the most expedient alterations in treatment recommendations were not implemented. Second, OS was the subject for the lead-time bias: i.e., those patients in whom the NLST was initiated following the receival of the CGP results, initiated the treatment later as opposed to patients in whom the NLST was started before the CGP results became available. Finally, higher proportion of patients in whom the treatment was initiated before receival of the CGP results appeared to harbor an ALK resistant mutation, which might have an impact on outcomes as well. The lack of the ability to demonstrate an impact of CGP on oncological outcomes remains the most significant limitation of our study, along with the small sample size. Another important study limitation is its non-randomized design allowing patient selection for immediate versus postponed treatment initiation based on the tempo of the disease.

Overall, the study has demonstrated the feasibility and the significant impact of the CGP on the decision-making process in ALK-rearranged aNSCLC following failure of 2^nd^/3^rd^-generation ALK TKIs. It remains to be seen whether such strategy affects oncological outcomes.

## Data Availability Statement

The datasets presented in this study can be found in online repositories. The names of the repository/repositories and accession number(s) can be found in the article/**Supplementary Material**.

## Ethics Statement

The studies involving human participants were reviewed and approved by the Helsinky ethics committee of the Rabin Medical Center (RMC), approval number: 0561-19-RMC. Written informed consent for participation was not required for this study in accordance with the national legislation and the institutional requirements.

## Author Contributions

Credit statement conceptualization: ED, AR, and AO. Methodology: ED, AR, and AO. Validation: ED. Formal analysis: TS. Investigation: ED. Resources: ED, AR, AO, LH, JD, DU, WK, AC, MM, AZ, JB, NR, SG, CO, AA, NP, and TS. Data Curation: ED. Writing - original draft: ED, AR, AO, and LH. Writing - review and editing: ED, AR, AO, LH, JD, DU, WK, AC, MM, AZ, JB, NR, SG, CO, AA, NP, and TS. Visualization: ED and LH. Supervision: ED. Project administration: ED. All authors contributed to the article and approved the submitted version.

## Conflict of Interest

Author TS was employed by the company Statistical Consulting Unit. Disclosure (all outside of the submitted work): AR reported personal fees from Roche, Astra Zeneca, Merck Sharpe & Dohme, Novartis, Takeda, Elli Lilly, support for attending meetings from Bristol Myers Squibb, Roche, Boehringer Ingelheim. AO reported advisory fees from Merck Sharpe & Dohme, Bristol Myers Squibb, Roche, Astra Zeneca, Novartis, Boehringer Ingelheim. Damien Urban reported personal and consulting fees from Roche, Merck Sharpe & Dohme, Takeda, Astra Zeneca, Rhenium Oncotest, Bristol Myers Squibb. MM reported consulting fees from Boehringer Ingelheim, Roche, Astra Zeneca, MSD, BMS, Abbvie, Takeda, Pomicell. AZ reported grants from Bristol Myers Squibb, personal fees from Roche, Merck Sharpe & Dohme, Bristol Myers Squibb, Astra Zeneca, Takeda. JB reported grants and personal fees from Merck Sharpe & Dohme, Bristol Myers Squibb, Astra Zeneca, Roche, Abbvie, Takeda, OncoHost, ImmuneAI, Bayer, Novartis. AA reported research funding from Bristol Myers Squibb, personal and consulting fees from Bristol Myers Squibb, Roche, Pfizer, Astra Zeneca, Takeda, Novartis. NP reported research funding from Bristol-Myers Squibb, Eli Lilly, Foundation Medicine, Gaurdant360, Merk, MSD, Novartis, NovellusDx, Pfizer, Roche, Takeda, IP: Volatile Organic Compounds For Detecting Cell Dysplasia And Genetic Alterations Associated With Lung Cancer, WO2012023138; Breath Analysis of Pulmonary Nodules, US20130150261 A1; Apparatus for treating a target site of a body, WO/2015/059646 - all outside of the submitted work. ED reported grants from Astra Zeneca, Boehringer Ingelheim, personal fees from Boehringer Ingelheim, Roche, Astra Zeneca, Pfizer, Merck Sharpe & Dohme, Bristol Myers Squibb, Novartis, Takeda, Sanofi, Merck Serono, Medison Pharma, Janssen Israel- all outside of the submitted work.

The remaining authors declare that the research was conducted in the absence of any commercial or financial relationships that could be construed as a potential conflict of interest.

## Publisher’s Note

All claims expressed in this article are solely those of the authors and do not necessarily represent those of their affiliated organizations, or those of the publisher, the editors and the reviewers. Any product that may be evaluated in this article, or claim that may be made by its manufacturer, is not guaranteed or endorsed by the publisher.

## Abbreviations

Adenoca, adenocarcinoma; ALK, anaplastic kinase lymphoma; (a)NSCLC, advanced non-small cell lung cancer; CDK4/6, cyclin-dependent kinase 4/6; CDKN2A/B, cyclin dependent kinase inhibitor 2A/B; CGP, comprehensive genomic profiling; CI, confidence interval; c-met, tyrosine-protein kinase Met; ECOG PS - Eastern Cooperative Oncology Group performance status; EGFR, epidermal growth factor receptor; gen., generation; IASLC, International Association for the Study of Lung Cancer; IGF-1R, insulin-like growth factor 1 receptor; IQR, Interquartile range; KIT, KIT proto-oncogene; (m)OS, (median) overall survival; (m)TTD, (median) time-to-treatment discontinuation; NA, not available/not applicable; NGS, next-generation sequencing, NLST, next-line systemic treatment; NR, not reached; ROS1, ROS proto-oncogene 1; SRC, proto-oncogene SRC; TAT, turnaround time; TKI, tyrosine kinase inhibitor.
