# Supplementary material for: The Impact of Comprehensive Genomic Profiling (CGP) on the Decision-Making Process in the Treatment of ALK-Rearranged Advanced Non-Small Cell Lung Cancer (aNSCLC) After Failure of 2nd/3rd-Generation ALK Tyrosine Kinase Inhibitors (TKIs)
Source: Front Oncol. 2022 May 13;12:874712. doi: 10.3389/fonc.2022.874712 (PMC9137396; doi:10.3389/fonc.2022.874712)
Supplement: Supplementary file 1 [file Table_1.docx]

**Supplementary Document S1. Questionnaire.**

*Abbreviations: ALK - anaplastic kinase lymphoma; aNSCLC - advanced non-small cell lung cancer; CGP - comprehensive genomic profiling.*

**Step 1**

1. Physician's name
2. Patient’s code number
3. Date of filling step 1 of the questionnaire
4. aNSCLC ALK-positive patient, treatment failure of one of the following (multiple choices are permitted):
   1. Crizotinib
   2. Ceritinib
   3. Alectinib
   4. Brigatinib
   5. Ensartinib
5. Recommended systemic treatment **before** receiving CGP results:
   1. Brigatinib
   2. Lorlatinib
   3. Ceritinib
   4. Alectinib
   5. Ensartinib
   6. Platinum-based chemotherapy
   7. Immunotherapy
   8. Carboplatin-paclitaxel-Bevacizumab-Atezolizumab combination

**Step 2**

1. Date of filling step 2 of the questionnaire
2. Recommended systemic treatment **after** receiving CGP results:
   1. Brigatinib
   2. Lorlatinib
   3. Ceritinib
   4. Alectinib
   5. Ensartinib
   6. Platinum-based chemotherapy
   7. Immunotherapy
   8. Carboplatin-paclitaxel-Bevacizumab-Atezolizumab combination

Please attach Foundation Liquid results, including test date, identifying patient details should be removed.
